# The Effect of Pairing Individuals With Different Social Skills on Interpersonal Motor Coordination

**DOI:** 10.3389/fpsyg.2018.01708

**Published:** 2018-09-21

**Authors:** Kae Mukai, Akito Miura, Kazutoshi Kudo, Seijiro Tsutsui

**Affiliations:** ^1^Department of Life Science, Graduate School of Arts and Sciences, The University of Tokyo, Tokyo, Japan; ^2^Graduate School of Interdisciplinary Information Studies, The University of Tokyo, Tokyo, Japan; ^3^The Faculty of Wellness, Tokai Gakuen University, Aichi, Japan

**Keywords:** interpersonal coordination, bimanual coordination, joint action, role determination, autism-spectrum quotient

## Abstract

Previous studies have demonstrated that combining individuals with different social skills affects performance in rhythmic interpersonal motor coordination, with individuals with lower social skills, such as individuals with autism spectrum disorder or schizophrenia, being found to follow the actions of partners with higher social skills. In this study, we investigated whether this finding could be generalized among pairs of individuals without disability. To perform this, we applied an interpersonal motor coordination task that required participants to perform rhythmic movements featuring an interpersonal relative phase pattern of 90°. We did not assign the two roles (i.e., the preceding and following roles) to the participants, meaning they were forced to determine which roles to adopt by observing each other’s movements, without verbal communication. Individual social skills were measured using the autism-spectrum quotient (AQ). We found that pairs of participants with widely differing AQ scores performed better than did pairs with similar AQ scores. Most notably, the participants with higher AQ scores tended to precede their partners in the present task, which is the opposite result to that reported in previous studies. Our findings suggest that paring individuals without disability according to their social skills influences their interpersonal coordination performance in tasks wherein they must determine the preceding and following roles themselves.

## Introduction

Interpersonal motor coordination (IMC) plays an important role in successful social interaction. Recent research has demonstrated that IMC and social cognitive processes interact with each other. For example, it has been determined that developing motor synchrony with others enhances social bonds, such as by increasing rapport (Bernieri, 1988; Bernieri et al., 1994, 1996), connectedness (Hove and Risen, 2009; Miles et al., 2009; Raddish et al., 2013), and likability (Launy et al., 2014). On the other hand, how an individual feels about others also affects their IMC with these people (Zhao et al., 2015, 2017). The dynamic systems approach is a useful tool for investigating this interaction between social characteristics and IMC (Schmidt et al., 1994, 2011; Schmidt and O’Brien, 1997; Miles et al., 2009; Varlet et al., 2012; Zhao et al., 2015, 2017; Fitzpatrick et al., 2016), and in this study, we applied this approach to investigate this relationship. In particular, we focused on how pairing individuals with differing social cognitive functions affects their IMC.

Compared with previous research on the individual behavioral characteristics of individuals with low social skills, such as individuals with autism spectrum disorder (ASD) (Klin et al., 2002; Chawarska et al., 2013; Martin et al., 2018), little is known about the effect pairing individuals with different social cognitive functions has on their IMC. Schmidt et al. (1994) were the first to report the combination effect in terms of social skills. In their study, the participants were instructed to maintain an anti-phase pattern while performing a pendulum-swinging task. Consequently, it was found that, among three groups of pairs, which were assigned based on the individuals’ degree of social skills (i.e., low-low pairs, high-low pairs, and high-high pairs), the high-low pairs were able to maintain the anti-phase pattern longest. The researchers suggested that this was because in the high-low pairs the preceding and following roles were easily determined, with the individual with higher social skills preceding and the individual with lower social skills following around the anti-phase pattern. Similar findings have been reported in terms of individuals with schizophrenia (Varlet et al., 2012) and with ASD (Fitzpatrick et al., 2016), where it was found that such individuals with schizophrenia or ASD never lead their partners in IMC when swinging a pendulum. The findings of these previous studies suggest combining individuals with different social cognitive functions affects IMC in terms of role determination.

In order to further progress understanding of the effect pairing individuals with different social cognitive functions has on IMC, in this study we applied a task in which participants created interpersonal relative phase patterns of 90° without engaging in verbal communication. Previous studies regarding this combination effect (Schmidt et al., 1994; Varlet et al., 2012; Fitzpatrick et al., 2016) have used in-phase or anti-phase patterns, which can be accomplished without the pair being required to determine who precedes and who follows. In other words, the participants did not consciously intend to precede or follow their partners, and it is speculated that they did not even notice they were preceding or following their partners. In contrast, in a task that requires participants to create interpersonal relative phase patterns of 90°, they must determine the roles (i.e., preceding or following) during the task. We assumed that in such a situation the degree of social skill would be critical for determining roles and the consequent IMC, because the participants would be forced to visualize and intuit whether their partner intended to precede or follow by observing the partners’ movement (Curioni et al., 2017).

The abovementioned previous studies on the combination effect (Schmidt et al., 1994; Varlet et al., 2012; Fitzpatrick et al., 2016) recruited sets of participants from two extremes in terms of social skill. For example, Schmidt et al. (1994) recruited individuals who scored in the upper and lower quartiles of a shortened version of the Social Skills Inventory (Riggio, 1986) among 271 university students who completed it. Meanwhile, Varlet et al. (2012) and Fitzpatrick et al. (2016) recruited individuals without disability and individuals who had been diagnosed with schizophrenia or ASD, respectively. Thus, it has not been investigated whether this combination effect can be found in individuals whose social skills lie between these two extremes. Consequently, in the present study, we investigate whether this combination effect can be generalized to individuals who have not been diagnosed as having a disorder.

In order to investigate the combination effect of social skills, we should also consider individual motor skill. The task we used in this study was expected to be difficult because a relative phase pattern of 90° is reported to be easily entrained into an in-phase or anti-phase pattern (Schmidt et al., 1990; Zivotofsly and Hausdroff, 2007). In sports such as tennis and badminton, if at least one of the two players is good, the pair can continue a long rally, irrespective of their social skills. Similarly, we assumed that if there is at least one individual with high motor skill in a pair, the pair would show high performance in IMC. Thus, we evaluated individual motor skill using a bimanual coordination task in which participants created relative phase patterns of 90° with both hands. This is because the same dynamic principle governs both bimanual (Haken et al., 1985; Zanone and Kelso, 1992) and IMC (Schmidt et al., 1990), and it was assumed that those who are good at bimanual coordination would show enhanced performance in the similar IMC task.

Thus, the purpose of this research was to investigate whether the effect pairing individuals with different social skills has on their IMC can be generalized to individuals without disability. In Experiment 1, participants performed a bimanual coordination task through which their individual motor skills were evaluated. Then, in Experiment 2, the same participants performed an IMC task in which they were asked to create interpersonal relative phase patterns of 90°. We measured social skill using the autism-spectrum quotient (AQ) (Baron-Cohen et al., 2001). Further, we hypothesized that pairs composed of individuals whose AQ scores differed to a great extent would show higher IMC performance, because in such pairs the role determination would be achieved easily, as suggested in previous research (Schmidt et al., 1994; Varlet et al., 2012; Fitzpatrick et al., 2016).

## Materials and Methods

### Participants

Twenty-three undergraduate students who are randomly selected from a university student population (12 males and 11 females), ranging in age from 19 to 21 years, participated in this study. In Experiment 1, all of the participants performed an intrapersonal bimanual coordination task. In Experiment 2, a total of 19 pairs were created from the 23 participants, and the IMC task was performed. Because no previous studies have been done on this topic and the effect size was unknown, we conducted a *post hoc* power analysis for a correlation analysis between the IMC performance value and the AQ ratio (see below), which revealed a power of 0.68. Twelve participants conducted Experiment 2 only once, seven participants performed it twice, and four participants performed it three times, (this was facilitated by changing partners between tests). There were nine same-gender pairs (four male pairs and five female pairs), and 10 different-gender pairs. All participants knew each other before the experiment. We fully explained the experiment details to all the participants in accordance with Declaration of Helsinki and obtained oral informed consent from them before execution of the experiment. These procedures were approved by the Internal Review Board at the Aichi University of Education.

### Apparatus

**FIGURE 1:**
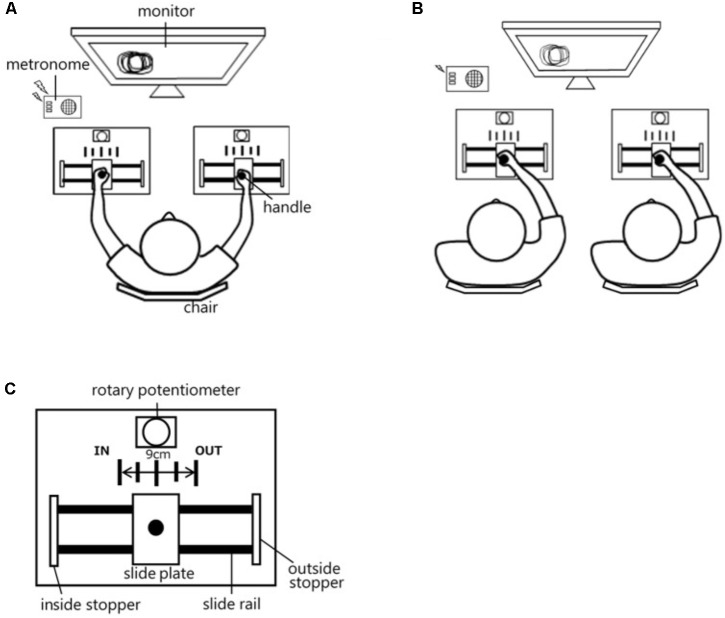
Schematic diagrams of Experiment 1 **(A)**, and Experiment 2 **(B)**. Detail drawing of the experimental equipment **(C)**.

In Experiment 1, the participants performed the bimanual coordination task by grasping two handles on a desk while sitting on a chair (**Figure 1A**). The height of the chair could be adjusted by the participant, if needed. Then, in Experiment 2, the pairs performed the interpersonal coordination task by grasping one handle with their dominant hand while their partner grasped the other handle with their dominant hand (**Figure 1B**). The handles were installed upright on plates resting on slide rails. “IN” and “OUT” indications were provided on the metal plates to indicate the range the participants could move the handles (**Figure 1C**). The distance between “IN” and “OUT” indication was 9 cm, and the movable range of the handles was 16 cm. The motion of the handles was measured by rotary potentiometers that were connected to the handles by wires. A monitor (height: 11.8 cm, width: 12.1 cm) was installed in front of the participants in order to provide real-time feedback on the handle motion. On the monitor display, a movement trajectory of 10 s (one trial duration) was depicted in real time on a phase plane that consisted of right handle displacement (horizontal axis) and left handle displacement (vertical axis). When the participants moved the handles at a relative phase pattern of 90° with identical movement amplitudes, a perfect circle was drawn on the monitor. When participants moved the handles in-phase, a straight line with a slope of -1 was drawn on the phase plane, and when the participants moved the handles in anti-phase, a straight line with a slope of 1 was drawn on the phase plane.

### Procedure

#### Experiment 1: Intrapersonal Bimanual Coordination Task

First, all of the participants performed Experiment 1. Then, on another day, they performed Experiment 2. In Experiment 1, we instructed the participants to perform periodic movements with both hands, creating a bimanual relative phase pattern of 90°, following a frequency specified by metronome beats. The frequency of the metronome was 2.5 Hz. They were instructed to synchronize one period of movement with two beats, so the movement frequency became 1.25 Hz. The phase of movement that was coordinated with the metronome beat was not specified. They were then requested to try to draw a circle on the monitor^1^. The duration of each trial was 10 s. The trials were started and ended by the experimenter’s oral direction. The participants performed a total of 135 trials (three sets of 15 trials per day for three consecutive days).

#### Experiment 2: Interpersonal Coordination Task

In Experiment 2, the participants of Experiment 1 were randomly assigned into pairs. Upon entering the laboratory, the participants were prohibited from talking to each other; they sat in a chair facing a desk on which the apparatus was placed. They were instructed to perform a periodic movement, at a frequency specified by metronome beats, with their dominant hands to create an interpersonal relative phase pattern of 90°. The frequency of the metronome and the instructions provided were the same as those in Experiment 1. Relative phase patterns of 90° could be achieved by drawing a circle on the display or by observing the partner’s hand movements. Both were allowed; however, the participants were instructed not to look at their partner’s face or to make eye contact. One trial duration was 10 s. The trial was started and ended by the experimenter’s vocal instruction. Each pair continued the experiment until they could perform the task successfully. The criterion for a successful trial was a mean relative phase of between 80° and 100° or between -100° and -80°. If the pair achieved two consecutive successful trials, the experiment ended. The upper limit of the number of trials was 80.

#### Questionnaire for Social Skills

Between Experiments 1 and 2, the participants were asked to complete the AQ questionnaire (Baron-Cohen et al., 2001, 2006). Specifically, the Japanese version of the AQ, which has previously been verified, was used (Wakabayashi et al., 2004, 2007). The questionnaire consists of five subscales (social skills, switching attention, attention to details, communication, and imagination) with 10 questions in each subscale. Participants answered this questionnaire through compulsory selection (four cases: “definitely agree,” “slightly agree,” “slightly disagree,” and “definitely disagree”). Thus, the score range for this questionnaire was 0 to 50 points. When the participants answered items that related to autism spectrum tendency, one point was given for each. “Definitely agree” and “slightly agree,” and “slightly disagree” and “definitely disagree” were considered as providing the same score, respectively. The higher the score, the higher the autism spectrum tendency.

### Analysis

In both Experiment 1 and 2, we evaluated coordination performance by counting the number of trials until two consecutive successful trials were performed. We defined a successful trial as one in which the mean relative phase lay between 80° and 100° or between -100° and -80°. The relative phase of the two handles was calculated using point estimates. First, we converted one period (i.e., left peak to left peak duration) of the left handle movement into 360°; then, we converted the time when the right handle reached the left peak into relative phase within the 360°. The relative phase values were converted into between -180° and 180°.

We calculated the ratio of AQ score for both participants in order to examine the relationship between the ratio of social skills and IMC performance. Specifically, the AQ ratio was computed using the following calculation formula:

$$\text{AQ ratio}=\frac{S_{L}}{S_{L}+S_{H}}$$

where S_L_ is lower AQ score and S_H_ is higher AQ score in the pair.

When the difference in AQ score between the two members of a pair is greater, the AQ ratio becomes smaller. We also calculated the Pearson product-moment correlation between the AQ ratio and the performance value in IMC. Each AQ subscale ratio was calculated in the same way as the AQ ratio.

We needed to consider the possibility that combinations of varying individual motor skills could affect the performance of IMC, irrespective of the combination of social skills. For example, a pair that includes at least one participant who was determined, through the bimanual coordination task, to have high motor skills could show enhanced performance in IMC. To investigate this, we summed performance value in the bimanual coordination task (i.e., number of trials until two consecutive successful trials) of the two participants in each pair and used it as a representative value for the pair. When a pair included a participant with high motor skill, this value was smaller. Then, we calculated the Pearson product-moment correlation between the summed performance value in the intrapersonal task and the performance value in IMC.

This study includes participants of both genders, which constitutes four pairs of males, five pairs of females and 10 different-gender pairs in IMC. To investigate the effect of these types of pairs on the pairwise variables (i.e., the performance value in IMC, summed performance value in the bimanual coordination task, AQ ratio, and AQ subscale ratio), we performed a one-way ANOVA on these variables.

## Results

### Performance Measures and AQ Score

The mean performance value for all participants in the bimanual coordination task (i.e., number of trials until two consecutive successful trials) was 33.2 ± 20.2 trials (mean ± SD). Meanwhile, the mean performance value in IMC task was 33.6 ± 20.8 trials.

The mean and SD of AQ score of all participants was 21.0 ± 6.1 (score range: 8–34). This AQ score is similar to the mean AQ score for the Japanese college students (20.7 ± 6.4) who participated in Wakabayashi et al. (2004). There was one participant who could be diagnosed with ASD (achieving an AQ score of 34 points); because the statistical significances in the correlation analyses were the same even if we excluded the data for this participant, we included this participant in the analysis.

To investigate the effect of types of pairs (pairs of males, pairs of females, and different-gender pairs) on the pairwise variables, we conducted a one-way ANOVA. As a result, we found that there was no significant difference in the performance value in IMC, the summed performance value in the bimanual coordination task, in AQ ratio, or in AQ subscale ratio (*ps* > 0.1) except for local detail ratio. Further, there was a marginally significant difference in local detail ratio [*F*(2,16) = 3.09, *p* = 0.07]. Thus, we performed the following analyses without classifying the three types of pairs.

### Correlation Between AQ Ratio and IMC Performance

**FIGURE 2:**
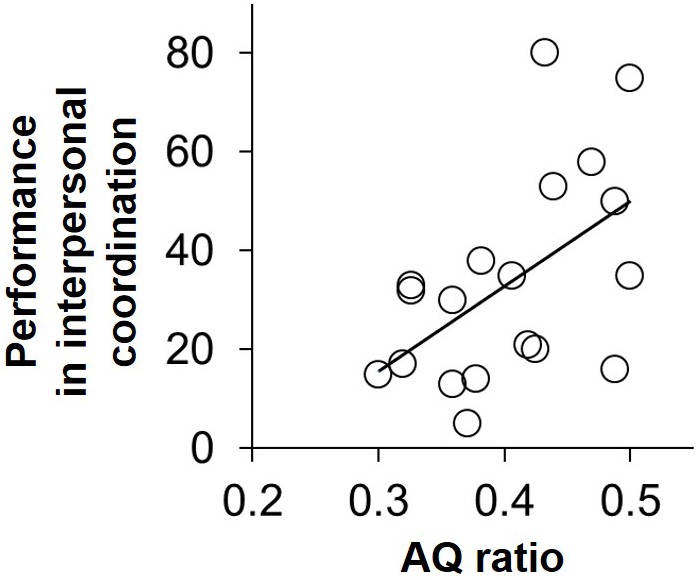
The scatter plot between AQ ratio and performance value in IMC. The Pearson product-moment correlation of these two variables was significant (*r* = 0.53, *p* = 0.01).

A scatter plot of the AQ ratio versus the performance value in IMC is shown in **Figure 2**. The Pearson product-moment correlation analysis of these two variables revealed a significant correlation (*r* = 0.53, *p* = 0.01). This result means that the pairs with lower AQ ratio (i.e., a pair composed of participants whose AQ scores differed from each other to a large extent) showed higher performance value in IMC than did the pairs with higher AQ ratios (i.e., pairs composed of participants with similar AQ scores).

### Correlation Between AQ Subscale Ratio and IMC Performance

**FIGURE 3:**
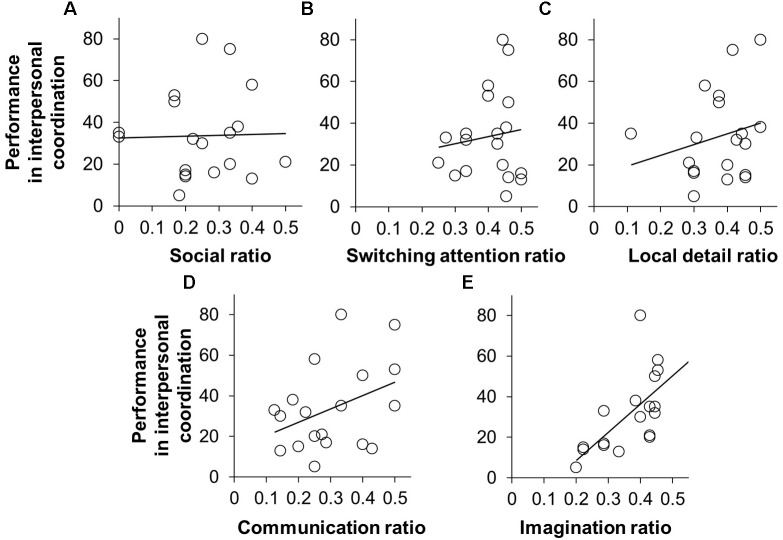
The scatter plots between AQ subscale ratio and the performance value in IMC; social skills ratio **(A)**, attention-switching ratio **(B)**, attention to detail **(C)**, communication ratio **(D)**, and imagination ratio **(E)**. The Pearson product-moment correlation between the imagination ratio and the IMC performance value was significant (*r* = 0.73, *p* = 0.001, after Bonferroni correction).

The scatter plots of each AQ subscale ratio versus the performance value in IMC are shown in **Figures 3A–E**. The Pearson product-moment correlation analysis of the performance value in the IMC task and each subscale ratio revealed no significant correlation in social ratio (*r* = -0.02, *p* = 1.00, after Bonferroni correction), attention-switching ratio (*r* = 0.12, *p* = 1.00, after Bonferroni correction), local details ratio (*r* = 0.23, *p* = 1.00, after Bonferroni correction), or communication ratio (*r* = 0.38, *p* = 0.45, after Bonferroni correction). Regarding imagination ratio, there was a significant correlation with the IMC performance value (*r* = 0.73, *p* = 0.001, after Bonferroni correction). These results mean that the pairs that were composed of participants whose scores for the imagination subscale differed to a greater extent showed better performance in IMC than did the pairs composed of participants with similar scores.

### Correlation Between Motor Skill and IMC Performance

**FIGURE 4:**
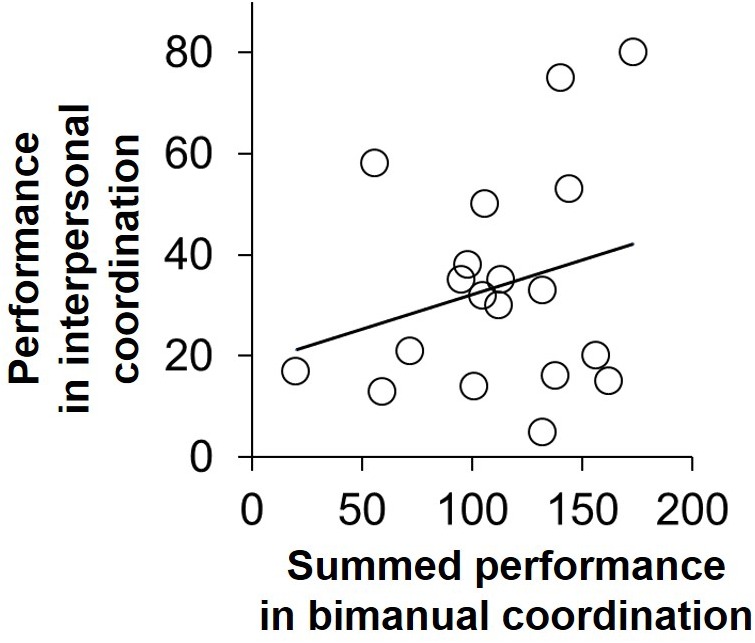
The scatter plot between summed performance value in the bimanual coordination task and the performance value in IMC. The Pearson product-moment correlation of these two variables was not significant (*r* = 0.25, *p* = 0.27).

The scatter plot of the summed performance value in the bimanual coordination task versus the performance value in IMC is shown in **Figure 4**. The Pearson product-moment correlation analysis of these two variables revealed no significant correlation (*r* = 0.25, *p* = 0.27). This result means that the combination of motor skills did not affect the IMC performance.

### Difference in Social Skills and Role in IMC

Considering the significant correlation between AQ ratio and the performance value in IMC, we further investigated whether the roles that the two participants played during IMC (i.e., preceding or following) were determined by the degree of difference in their social skills. The roles of the two participants in each pair were classified by the roles that were played in the two successful trials (i.e., the last two trials). By applying this criterion, the roles of one pair were found to be undeterminable because the roles differed over the last two successful trials; thus, we investigated the other 18 pairs. Consequently, we determined that in 13 of the 19 pairs, the participants with higher AQ score (i.e., the participants with low social skills) took the preceding role. We had originally hypothesized that if the AQ score did not affect the role, the number of pairs in which the participant with lower AQ score took the preceding role would be equal to that of the pairs in which the participant with higher AQ score took the preceding role. The chi-square test tends to reject this null hypothesis (*x^2^* = 5.64, *df* = 1, *p* = 0.05). This result means that participants with higher AQ scores tend to play a preceding role. We found a similar result with respect to imagination skill. In 13 of the 19 pairs, the participants with higher imagination score (i.e., the participants with low social skills) took a preceding role (*x*^2^ = 5.64, *df* = 1, *p* = 0.05).

## Discussion

In this study, we investigated whether the combination of social skills and of motor skills influenced IMC performance. Consequently, we found that pairs composed of participants whose social skills differed from each other to a relatively large extent showed better performance in IMC than did pairs composed of participants with similar social skills (**Figure 2**). Notably, imagination skill among the subscales of AQ score particularly affected IMC performance (**Figure 3**), and we confirmed that the combination of motor skills did not affect IMC performance (**Figure 4**). These findings provide additional evidence that social skill affects IMC, especially individuals with lower social skills do not necessarily show lower IMC performance in particular situations where roles should be determined. In other words, interpersonal performance can be determined by the interaction between the individuals, and not by the characteristics of just one individual.

Among the subscales of AQ score, only imagination skill ratio showed a significant correlation with the performance of IMC. The subscale of the imagination skill evaluates the skills to read and imagine others’ intent (Baron-Cohen et al., 2001, 2006). This means that a pair composed of a participant who can effectively imagine the intentions of their partner and a participant who cannot do so perform better in the present IMC. In addition, we found that the participants with higher AQ scores in a pair tended to precede their partners, and participants with lower AQ scores tended to follow their partners. The same result was found for the subscale scores regarding imagination skill. These findings can be interpreted as follows: a participant with low social skill (i.e., higher AQ score and higher imagination score) cannot read or intuit the intentions of their partner. Such people move the handle without thinking of their partners’ actions. Meanwhile, a participant with high social skill can read and imagine the intentions of their partner and move the handle accordingly. Thus, in pairs that include participants with high and low social skills, the preceding and following roles can be determined at an early stage in the task, which leads to enhanced IMC performance.

It is noteworthy that the role adoption in the present study is opposite to that reported in previous studies (Schmidt et al., 1994; Varlet et al., 2012; Fitzpatrick et al., 2016), in which individuals with higher social skills preceded and individuals with low social skill followed. There are two differences between these studies and the present research: the degree of social skills of the participants and the experimental task in question. For example, Fitzpatrick et al. (2016) investigated combining individuals without disability and those with ASD by using an in-phase and anti-phase task, consequently finding that individuals with ASD followed the individuals without disability. In the present research, there was one participant whose AQ score was 34 points, meaning they could be diagnosed with ASD; however, this participant preceded in IMC with individuals without disability. Thus, the inconsistency may not be due to the degree of social skills, but may be due to the specificity of the present task. This should be confirmed in future research.

This study has some limitations. First, we assessed the social skills by using only the self-report AQ questionnaire. In the future, a more objective evaluation should be done to increase the accuracy of the assessment of individual social skills. Second, the sample size was relatively small for the effect size of this experiment, which was determined via a *post hoc* power analysis (with a power of 0.68). As we now have a baseline effect size, we should increase the sample size in a future study. Finally, it remains unknown whether our findings can be generalized to other populations (e.g., individuals with social disorders) or to other IMC tasks. We intend to confirm this in a future controlled experiment.

## Conclusion

We investigated whether a combination of social skills influences IMC when the preceding and following roles must be determined by observing partners’ movements. Consequently, we found that the pairs composed of participants whose social skills differed from each other to a great extent exhibited higher IMC performance than did pairs composed of individuals with similar social skills. We also confirmed that participants with lower social skills than their partners tended to precede their partners in the task. These findings provide additional evidence that social skill affects IMC performance, especially in terms of role determination. Although the disabilities of individuals with lower social skills have frequently been reported, this study suggests that these individuals can perform better in certain situations—that is, where the roles are determined by considering a pair’s combination of social skills. We must emphasize that these findings were observed using an original task where participants determined on their own whether to lead or follow their partner. In the future, we should investigate whether or not these findings can be generalized to other populations and to other IMC tasks.

## Author Contributions

KM and ST contributed to the conception and design of the work. KM performed the data acquisition. KM, AM, KK, and ST contributed to the data analysis, interpretation of the data, and drafting of the manuscript.

## Conflict of Interest Statement

The authors declare that the research was conducted in the absence of any commercial or financial relationships that could be construed as a potential conflict of interest.

## Footnotes

1 In Experiment 1, feedback on the monitor was given in real time for half of the participants and immediately after the trial for the other half. This was because the purpose of Experiment 1 initially differed. However, because we confirmed that the performance value was not significantly different between these groups, we combined these groups and all of the participants performed Experiment 2 together.
